# A Complete Sustained Response of Advanced Non-Small-Cell Lung Cancer After Immune Checkpoint Inhibitor, Radiotherapy, and Chemotherapy

**DOI:** 10.7759/cureus.32585

**Published:** 2022-12-16

**Authors:** Isabel Vilas-Boas, Inês Moreira, Ana Rodrigues

**Affiliations:** 1 Medical Oncology, Instituto Português de Oncologia do Porto Francisco Gentil, EPE, Porto, PRT

**Keywords:** complete response, palliative treatment, synergic mechanisms, radiotherapy, non-small cell lung cancer, immunotherapy, chemotherapy

## Abstract

Lung cancer is the leading cause of cancer-related mortality worldwide. The treatment of advanced lung cancer is improving with the development of new treatments like immune checkpoint inhibitors (ICIs) and various molecular targeted agents, which have extended overall survival (OS). However, complete remissions remain rare. The efficacy of chemotherapy is modest, which makes a complete sustained response very unlikely, especially when compared with more recent options.

In this article, we report a stage IV non-small-cell lung cancer (SCLC) that achieved a complete response in 2018 with chemotherapy (cisplatin and paclitaxel) after pembrolizumab and after the patient had received radiotherapy for superior vena cava syndrome (SVCS). The patient remains in complete response as of October 2022. We hypothesized that the overlap between circulating anti-PD-1, radiotherapy, and cytotoxic agents could explain this outcome.

## Introduction

Lung cancer is the leading cause of cancer-related mortality worldwide, with a five-year survival rate of only 18-21% considering all cases [1,2] and only 6% for advanced disease, according to Surveillance, Epidemiology, and End Results (SEER). In Europe, in 2018, lung cancer was the most common cause of death from cancer in men (267,000 deaths, 24.8%) and the second most common in women (121,000 deaths, 14.2%) after breast cancer [1,3].

Small-cell lung cancer (SCLC; approximately 15% of cases) and non-small-cell lung cancer (NSCLC) are the two main types of lung cancer. Most patients (85%) present with an advanced stage at diagnosis and can receive palliative systemic treatment or the best supportive care based on their performance status. The efficacy of chemotherapy is modest in these patients. However, the treatment of advanced lung cancer improved significantly with the development of new treatment alternatives like immune checkpoint inhibitors (ICIs) and various molecular targeted agents. ICIs, particularly inhibitors of the PD-1 axis, have modified the management of NSCLC over the last ten years [4]. ICIs improve overall survival (OS) compared to chemotherapy when used as first-line therapy for patients whose tumors express PD-L1 on at least 50% of cells [5]. Also, combining ICIs with chemotherapy has improved survival in patients with both squamous and non-squamous NSCLC [6,7].

The prevalence of complete remissions is not estimated, and the specific features of long-responders with advanced NSCLC have not been elucidated.

In this article, we report a stage IV non-small-cell lung cancer case that achieved a complete response with chemotherapy in a second-line setting after the failure of immunotherapy.

## Case presentation

**Figure 1:**
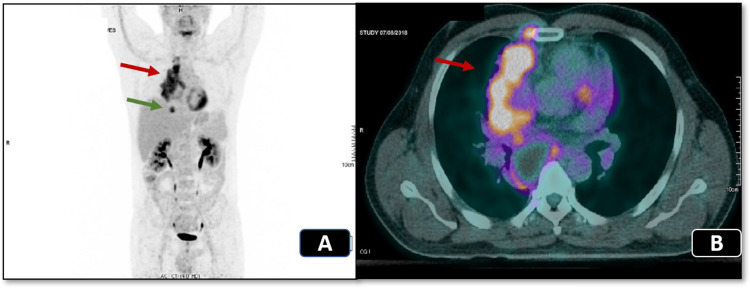
PET CT in August 2018 (A - coronal plane; B - transversal plane) showing the paramediastinal mass (red arrow) and the epiphrenic lymph node (green arrow).

We present the case of a 45-year-old nonsmoker with no comorbidities and a performance status of 0, who worked in a tree-felling and cutting business. He was referred to our oncological institution in June 2018 due to a lung mass found because he complained of dyspnea. A thoracoabdominal computed tomography (CT) showed a paramediastinal mass (100 mm × 86 mm × 46 mm) in the right superior lobe involving half of the ascending thoracic aorta´s circumference. The tumor was in contact with the pleura, the right pulmonary artery, and very close to the superior vena cava. No evidence of lung or pericardial effusion was found, nor was adrenal or hepatic metastasis. The patient had a cerebral CT that was negative for secondary lesions. PET CT (images A and B of Figure 1) showed 18-FDG uptake of the sizeable paramediastinal mass in the right lung, which was compatible with a malignant lesion. There was hypermetabolic activity in the right hilar, paratracheal, subcarinal, epiphrenic, and right internal mammary lymph nodes. These lesions represented lymph node metastasis.

A transthoracic biopsy revealed a non-small-cell lung carcinoma. Immunoexpression was positive for CK8/18, CK7 (weak and focal), P63 (focal and low intensity), and CD10, and negative for CK20, TTF1, and CD45. Chromogranin and synaptophysin were negative. The clinical stage of the neoplasia was T4N2M1b (stage IV due to non-regional lymph nodes), according to the AJCC Cancer Staging 8th edition. Next-generation sequencing (NGS) did not identify any potential molecular targets. More specifically, there were neither epidermal growth factor receptor (EGFR) mutations nor ROS1/ALK rearrangements. The immunohistochemical analysis with the monoclonal mouse anti-human PD-L1, Clone 22C3, revealed PDL1 expression in 90-100% of the cells.

The patient started pembrolizumab (200 mg intravenously every three weeks) in September 2018, and a CT after three cycles revealed stable disease (9.5 cm × 5.7 cm). He received five cycles until January 2019 with no documented toxicities. However, that month, the patient presented to the emergency department with cervical and facial edema. There were signs of collateral circulation in the left hemithorax. A CT angiography revealed a severe obstruction of the superior vena cava caused by the tumor, which had more significant dimensions than the previous exams. The tumor was 10.6 cm × 7.7 cm and invaded the right cardiac atria, the azygos vein, and the superior vena cava. The patient was hospitalized with the diagnosis of superior vena cava syndrome (SVCS). He initiated tinzaparin at 10.000 UI per day and received radiotherapy treatment (RT), 30 Grays divided into ten sessions, with a resolution of symptoms.

In February, the patient fully recovered and started second-line treatment with platinum-containing doublet chemotherapy (cisplatin 80 mg/m^2^ IV and paclitaxel 175 mg/m^2^ IV every three weeks). According to Response Evaluation Criteria In Solid Tumours (RECIST) 1.1 criteria, the thoracic CT after three cycles showed a partial response of the tumor (dimensions of 8.5 cm × 4.1 cm) and lymph nodes.

The patient received six cycles until May 2019 with good tolerance. The only toxicity documented was grade 1 neuropathy, according to the Common Terminology Criteria for Adverse Events (CTCAE) classification. He continued to work full-time in tree felling and cutting services during all his treatments.

**Figure 2:**
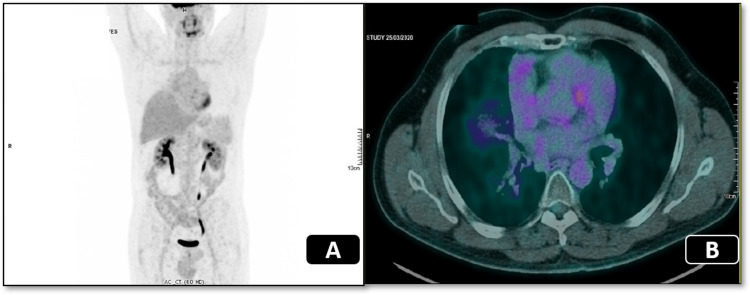
PET CT in March 2020 shows the tumor lesion disappearance (A - coronal plane; B - transversal plane).

The thorax CT of July 2019 revealed a stable tumor lesion with 8 cm × 4 cm, and the right lateral tracheal lymph nodes decreased by 1-2 mm. There was no evidence of superior vena cava extrinsic compression. Three months later, the patient remained asymptomatic and had a good performance status. There was no progression in the new CT in September 2019, which showed a tumor of 3.8 cm. This exam was repeated in December, and decreasing of the tumor was still noticeable, as the tumor presented a largest diameter of 3 cm. Since there was a progressive reduction of the tumor, a PET-FDG CT was requested in March 2020, and, remarkably, it showed no evidence of the tumor or other lesions with 18F-FDG hypermetabolic activity (images A and B of Figure 2). There was only a subtle FDG uptake in the right paramediastinal opacification and in the right tracheal lymph nodes, which were interpreted as residual lesion/inflammation. The last hospital appointment was in October 2022; the patient had no new symptoms, and the exams did not reveal disease progression. The patient will be monitored at our institution continuously.

## Discussion

In this article, we report a clinical case of a patient with advanced lung cancer who achieved a complete response after receiving chemotherapy in a second-line setting. This is an infrequent outcome, especially with chemotherapy. We assumed that some particularities in this treatment combination and schedule could explain this remarkable response.

In this case, pembrolizumab was expected to be the best treatment option according to the state-of-the-art [2,4]; therefore, early progression was not expected. Although there was no progression according to RECIST criteria (increasing was inferior to <20%), the patient experienced clinical progression (superior vena cava syndrome), and a change of treatment was necessary.

A possible explanation is that the patient experienced hyperprogressive disease, defined as rapid disease progression after initiation of immunotherapy, according to criteria that are not universally defined [5]. In NSCLC, the incidence of hyperprogression is 13.8-25.7% [6,7]. Patients with symptoms suggesting hyperprogressive disease should not receive further ICIs and should be switched to another potentially efficient treatment, such as chemotherapy, if the patient can tolerate it [5].

On the other hand, pseudoprogression, defined as an initial increase in the dimensions of cancer lesions due to the infiltration of tumoral tissue by immune cells, followed by a response to treatment [8], could be responsible for this case evolution. This phenomenon is not common, accounting for 3-5% of patients with NSCLC treated with anti-PD-1/PD-L1 [9,10]. Our patient could be responding to immunotherapy, and the SVCS is a consequence of the increased inflammatory response in the tumor. In that case, when the patient started chemotherapy as a second-line treatment after receiving first-line pembrolizumab and radiotherapy for the SVCS, an overlap of efficient drugs could have explained the excellent outcome. It is interesting to note that pseudoprogression seems to indicate a high likelihood of one-year survival [8,11], which is the case with our patient.

However, pseudoprogression and hyperprogression are more common in the first cycles of ICIs. In the case depicted, there was a stable disease after three cycles of pembrolizumab, making these phenomena more unlikely. It seems more plausible that the patient experienced progression under immunotherapy.

Some retrospective studies and case reports demonstrate an unexpectedly good response to chemotherapy following progression under immunotherapy [12,13], similar to the response that our patient had experienced. The hypothesis that immunotherapy and chemotherapy had a synergic effect was debated in those studies. Interestingly, chemotherapy has historically been considered immunosuppressive. However, it is now recognized that certain chemotherapy drugs can increase tumor immunity in many ways. Chemotherapy can induce immunogenic cell death - tumor cell lysis that generates new antigens that will be presented to the dendritic cells - and enhance tumor antigen presentation by upregulating the expression of tumor antigens or of the major histocompatibility complex class I molecules to which the antigens bind. Alternatively, chemotherapy may upregulate co-stimulatory molecules or downregulate co-inhibitory molecules, like PD-L1, expressed on the tumor cell surface and can also render tumor cells more sensitive to T cell-mediated lysis [14,15].

Others have also demonstrated the delayed synergic effect of anti-PD1 on subsequent cytotoxic therapy [16,17]. However, new studies with more significant cohorts must be performed to better understand this phenomenon and identify the best treatment sequence and potential responders.

Park et al. [18] presented a study that compared the objective response rates (ORRs) of salvage chemotherapy administered after immunotherapy with the ORRs of the ChT administered before immunotherapy. The results showed superior ORRs in patients receiving ChT after immunotherapy (53.4% vs. 34.9%, p = 0.03).

Finally, it is essential to note that our patient received RT seven days after the last administration of pembrolizumab. RT can induce increased antigenic expression on tumor cells (also by immunogenic cell death) and favor the release of pro-inflammatory chemokines, which promote the recruitment of effector CD8 and CD4 T cells [19,20]. Also, tumor cells that receive sublethal doses of radiation undergo phenotypic changes that enhance their susceptibility to immune effectors [20]. These could have potentiated the effect of pembrolizumab that was still in circulation (half-life of 12 to 27 days) [13], which could have influenced the outcome of the case presented.

## Conclusions

An interesting and remarkable complete response to advanced-stage lung cancer was depicted in this case report. An overlap among circulating anti-PD1, radiotherapy treatment, and cytotoxic agents could have contributed to improved treatment efficacy, which validates the new paradigm of oncology that the combination of different types of treatment leads to a better response due to synergic mechanisms.
